# Crowdbreaks: Tracking Health Trends Using Public Social Media Data and Crowdsourcing

**DOI:** 10.3389/fpubh.2019.00081

**Published:** 2019-04-12

**Authors:** Martin M. Müller, Marcel Salathé

**Affiliations:** ^1^Digital Epidemiology Lab, EPFL, Geneva, Switzerland; ^2^School of Life Sciences, EPFL, Lausanne, Switzerland; ^3^School of Computer and Communication Sciences, EPFL, Lausanne, Switzerland

**Keywords:** data mining, natural language processing (NLP), crowdsourcing, social media data, sentiment analysis (SA), vaccination, data stream analytics, machine learning

## Abstract

In the past decade, tracking health trends using social media data has shown great promise, due to a powerful combination of massive adoption of social media around the world, and increasingly potent hardware and software that enables us to work with these new big data streams. At the same time, many challenging problems have been identified. First, there is often a mismatch between how rapidly online data can change, and how rapidly algorithms are updated, which means that there is limited reusability for algorithms trained on past data as their performance decreases over time. Second, much of the work is focusing on specific issues during a specific past period in time, even though public health institutions would need flexible tools to assess multiple evolving situations in real time. Third, most tools providing such capabilities are proprietary systems with little algorithmic or data transparency, and thus little buy-in from the global public health and research community. Here, we introduce Crowdbreaks, an open platform which allows tracking of health trends by making use of continuous crowdsourced labeling of public social media content. The system is built in a way which automatizes the typical workflow from data collection, filtering, labeling and training of machine learning classifiers and therefore can greatly accelerate the research process in the public health domain. This work describes the technical aspects of the platform, thereby covering the functionalities at its current state and exploring its future use cases and extensions.

## 1. Introduction

In the past years, data derived from public social media has been successfully used for capturing diverse trends about health and disease-related issues, such as flu symptoms, sentiments toward vaccination, allergies, and many others (1–5). Most of these approaches are based on natural language processing (NLP) and share a common workflow. This workflow involves data collection, human annotation of a subset of this data, training of a supervised classifier, and subsequent analysis of the remaining data. The approach has proven promising in many cases, but it also shares a few shortcomings. A major drawback of this type of research process is that a model, which was trained on data from previous years, might not generalize well into the future. This issue, commonly known as concept drift (6), may not necessarily be only related to overfitting, but may simply be a consequence of how language and content, especially on the internet, evolve over time. A similar effect has been suggested to be the main reason for the increasing inaccuracy of Google Flu Trends (GFT), one of the most well-known flu surveillance systems in the past (7). After launching the platform in 2003, GFT's model had been retrained in 2009, which led to a significant improvement of its performance in the following years. However, during the influenza epidemic in 2012/13, the model's performance decreased again and overestimated the extent of the epidemic by a large margin. Shortly after, it was discontinued (8, 9).

Apart from the issue of model drift, a second issue associated with current NLP models is that the collection of large amounts of labeled data, usually through platforms such as Amazon Turk^1^ (MTurk), is very costly. Labeling a random subset of the collected social media data may be inefficient, as depending on the degree of filtering applied, large fractions of the collected data are possibly not relevant to the topic, and therefore have to be discarded.

Lastly, there is a growing interest in the public health field to capture more fine-grained categorizations of trends, opinions or emotions. Such categorizations could allow to paint a more accurate picture of the nature of the health issue at hand. However, multi-class annotations of a large sample of data again exponentially increases costs.

Here, we introduce Crowdbreaks^2^, a platform targeted at tackling some of these issues. Crowdbreaks allows the continuous labeling of public social media content in a crowdsourced way. The system is built in a way which allows algorithms to improve as more labeled data is collected. This work describes the functionalities of the platform at its current state as well as its possible use cases and extensions.

## 2. Similar Work

In recent years, a number of platforms have been launched which allow the public to contribute to solving a specific scientific problem. Among many others, examples of successful projects include the Zooniverse platform (formerly known as Galaxy Zoo) (10), Crowdcrafting (11), eBird (a platform for collecting ornithological data) (12), and FoldIt (a platform to solve protein folding structures) (13). Many of these projects have shown that citizen science can be used to help solve complex scientific problems. At the same time, there is a growing number of platforms which offer monetary compensations to workers for the fulfillment of microtasks (the most prominent example being MTurk). These platforms gain importance as the need for large amounts of labeled data for the training of supervised machine learning algorithms increases. Previous work focused mostly on efficiency improvement of large-scale human annotation of images, e.g., in the context of the ImageNet project (14). Most of these improvements include better ways to select *which* data to annotate, *how* to annotate (which is a UI specific problem) and what type of annotations (classes and subclasses) should be collected (15). Online task assignment algorithms have been suggested which may consider both label uncertainty as well as annotator uncertainty during the annotation process (16, 17). Results suggest that this allows for a more efficient training of algorithms. More recently, a crowd-based scientific image annotation platform called Quantius has been proposed, showing decreased analysis time and cost (18). To our knowledge, no similar work has been proposed with the regard to the human annotation of textual data, such as tweets.

## 3. Methods and Tools

Crowdbreaks is a platform which aims at automatizing the whole process from data collection (currently through Twitter), filtering, crowdsourced annotation and training of Machine Learning classifiers. Eventually these algorithms can help evaluate trends in health behaviors, such as vaccine hesitancy or the risk potential for disease outbreaks.

**Figure 1:**
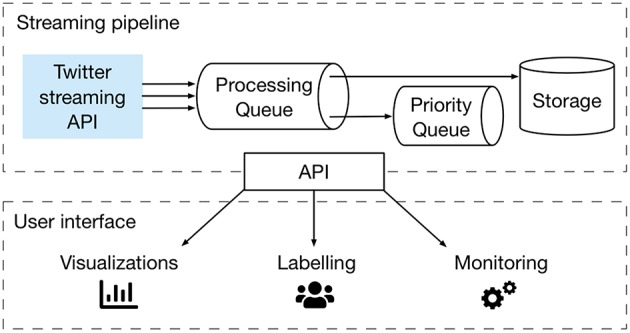
Overview of the architecture of the Crowdbreaks platform. The platform consists of a streaming pipeline (a message queueing system) and a user interface, linked through an API.

Crowdbreaks consists of a data collection pipeline^3^ (“streaming pipeline”) and a platform for the collection of labeled data^4^ (“user interface”), connected through an API (Application Programming Interface), as schematized in Figure 1.

### 3.1. Streaming Pipeline

Currently Crowdbreaks consumes data from the Twitter streaming API only, therefore the rest of this work will focus on tweets as the only data source. However, it could be extended to any textual data which can be collected in the form of a data stream through an API. The Twitter API allows for the filtering of tweets by a specific set of keywords in real-time. Tweets collected contain at least one exact match within certain fields of the tweet object. Incoming tweets are put on a background job queue for filtering, pre-processing, geo-tag enrichment, and annotation with metadata, such as estimated relevance or sentiment (more on this in section 5). After these processing steps, tweets are stored in a database. Based on a priority score (e.g., the uncertainty of a predicted label, see section 3.3.1) the tweet IDs are also pushed into a priority queue for subsequent labeling. Once the priority queue has reached a certain size, older items with low priority are removed from the queue and replaced with more recent items. Therefore, the queue keeps a pool of recent tweets which are prioritized for labeling. Once a tweet has been labeled, it is ensured that the same tweet will be labeled by a certain number of distinct users in order to reach a consensus.

### 3.2. User Interface

**Figure 2:**
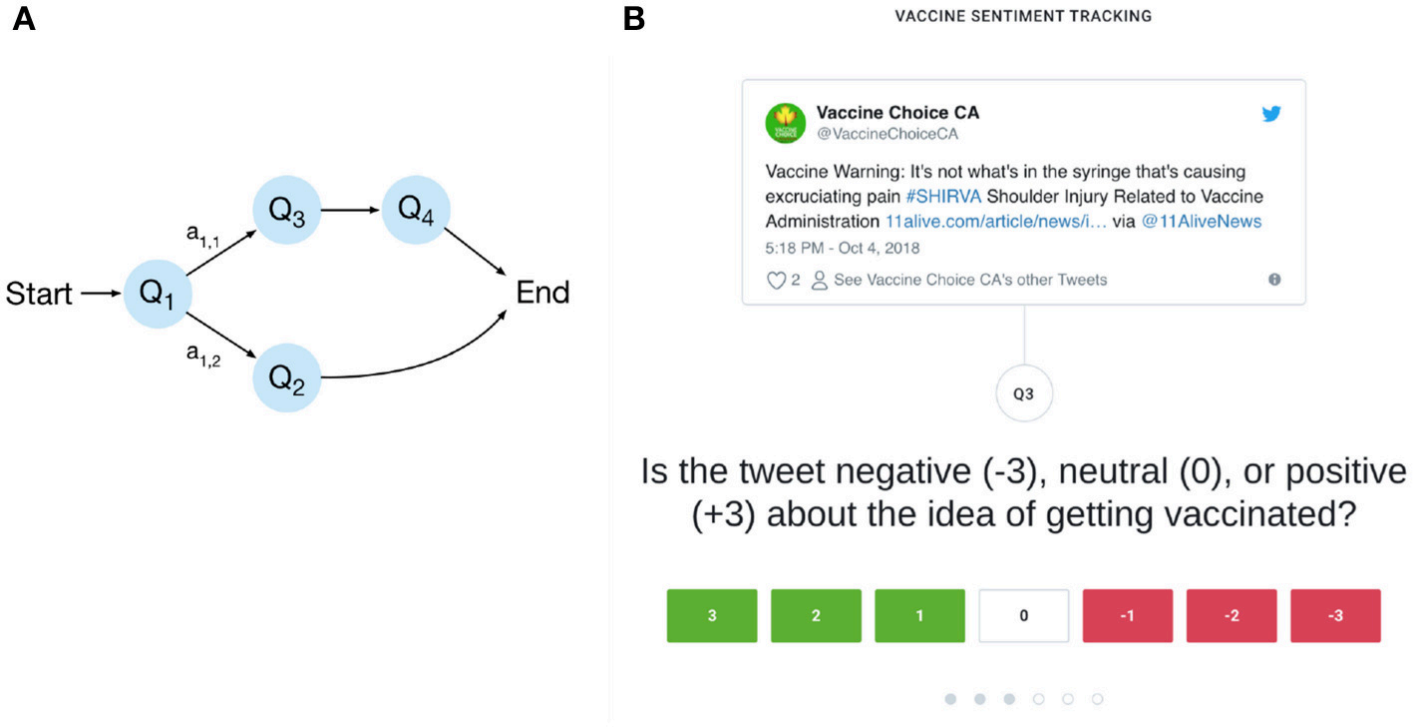
**(A)** An example of a question sequence. Questions are denoted by *Q*, answers by *a* and the arrows designate the possible transitions between questions. In the given example, different questions are reached depending on whether an annotator answers *Q*_1_ with *a*_1,1_ or *a*_1,2_ allowing for an efficient and fine-grained annotation of the data. **(B)** Screenshot of the annotation interface. Shown is a question for determining the vaccine sentiment of a tweet which has been deemed relevant to the topic.

The user interface allows labeling of tweets based on answering of a sequence of questions. Arbitrary question sequences can be defined, which allow the annotation of multiple classes and subclasses to a single tweet. Most commonly, different follow-up questions would be asked depending on the answers given previously, e.g., whether or not the tweet is relevant to the topic at hand (see Figures 2A,B). In the beginning of a question sequence an API call is made to the streaming pipeline to retrieve a new tweet ID from the priority queue (see section 3.1). Every question a user answers creates a new row in a database table, containing the respective user, tweet, question and answer IDs. After the user has successfully finished the question sequence the respective user ID is then added to a set, in order to ensure that the same tweet is not labeled multiple times by the same user.

Crowdbreaks supports multiple projects, each project may be connected to its own data stream from Twitter. New projects can be created through an admin interface, making it possible to control both the data collection, as well as to define project-specific question sequences. Eventually, visualizations, such as sentiment trends over time, may be presented to the public user, allowing the users to see the outcomes of their work. Crowdbreaks also features an integration of the question sequence interface with Amazon Turk, allowing the collection of labeled data through paid crowdworkers as an alternative to public users.

### 3.3. Sentiment Analysis

#### 3.3.1. Algorithms

In recent years, algorithms for sentiment analysis based on word embeddings have become increasingly more popular compared to traditional approaches which rely on manual feature engineering (19–21). Word embeddings give a high-dimensional vector representation of the input text, usually based on a pre-trained language model. Although these approaches may not consistently yield better results compared to traditional approaches, they allow for an easier automatization of the training workflow and are usually more generalizable to other problems. This is a desirable property in the context of Crowdbreaks, as it aims to further automatize this process and retrain classifiers automatically as more labeled data arrive. Furthermore, pre-trained word embeddings based on large Twitter corpora are available in different languages, which also make them interesting for following health trends in languages other than English (22). At its current state, the platform makes use of a baseline fastText classifier (21), which is trained on a small set of labeled data. FastText models are quickly re-trained and lead to small model sizes, making them suitable to be used in active learning production environments.

#### 3.3.2. Active Learning

**Figure 3:**
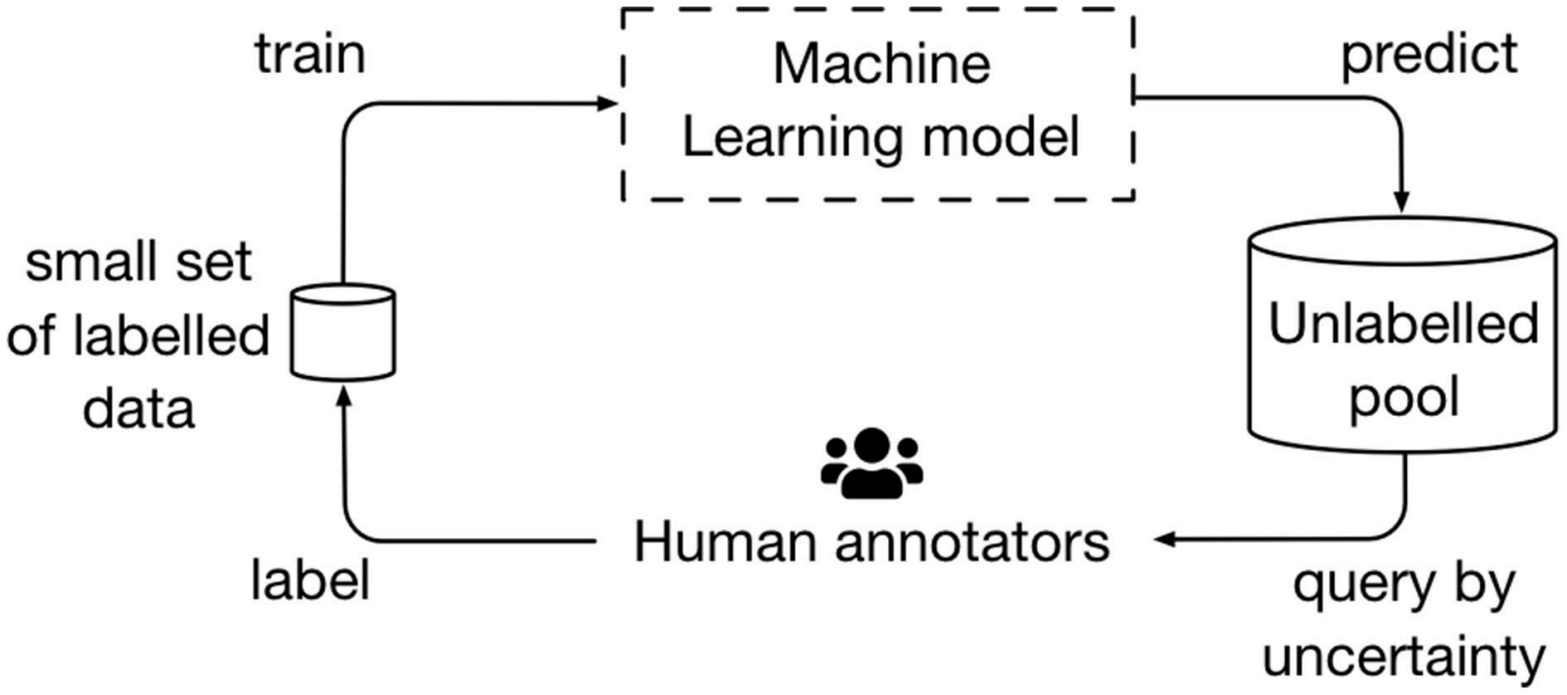
Crowdbreaks can be seen as an active learning framework which allows to improve algorithms as more labels are collected. In this example, an algorithm tries to learn sentiments from tweets and is given an initial small set of labeled data to be trained on. This algorithm may then be used to predict the labels and label uncertainty of newly collected tweets. Subsequently, tweets which the algorithm is most uncertain about will be presented to human annotators. As new labeled data is generated, the algorithm is retrained to further improve in performance.

Active learning frameworks have been proposed for a more efficient training of classifiers in the context of word embeddings (23, 24). These frameworks allow algorithms to be trained with a much smaller number of annotated data, compared to a standard supervised training workflow (see Figure 3). The query strategy, which is usually related to label uncertainty, is generally the critical component for the relative performance speed-up of these methods. In the context of Crowdbreaks, we are not only prioritizing data with higher label uncertainty, but also data which is more recent in time. Therefore, we are faced with a trade-off between exploration and exploitation with regard to label uncertainty and timeliness of data. Crowdbreaks can serve as a framework to explore these challenges and find the right balance.

### 3.4. Technologies Used

Crowdbreaks uses a Python Flask API to interface between the components of the streaming pipeline and the user interface. The streaming pipeline makes use of Redis for the message queuing of the processing queue as well as the priority queue (see Figure 1). Filtering and data processing, as well as NLP-related tasks are written in Python using the standard data analysis toolchain (numpy, scipy, nltk). Tweet objects are stored as flat files as well as in JSON format on Elasticsearch, which allows for an easier exploration and visualization of the data using Kibana. The user interface is built using Ruby on Rails with a postgres database backend in order to store the annotations, as well as user-related data.

All tools in the Crowdbreaks stack are open source and easy to deploy using Docker. The choice of tools was influenced by their long-term availability, community support and openness.

## 4. Results

The intensity, spread and effects of public opinion toward vaccination on social media and news sources has been explored in previous work (3, 25). Declines in vaccine confidence and boycotts of vaccination programs could sometimes be linked to disease outbreaks or set back efforts to eradicate certain diseases, such as polio or measles (26, 27). In particular, the potential benefits of real-time monitoring of vaccine sentiments as a tool for the improved planning of public health intervention programs has been highlighted (28–30). Tracking of such sentiments toward vaccines is a primary use case of Crowdbreaks.

**Figure 4:**
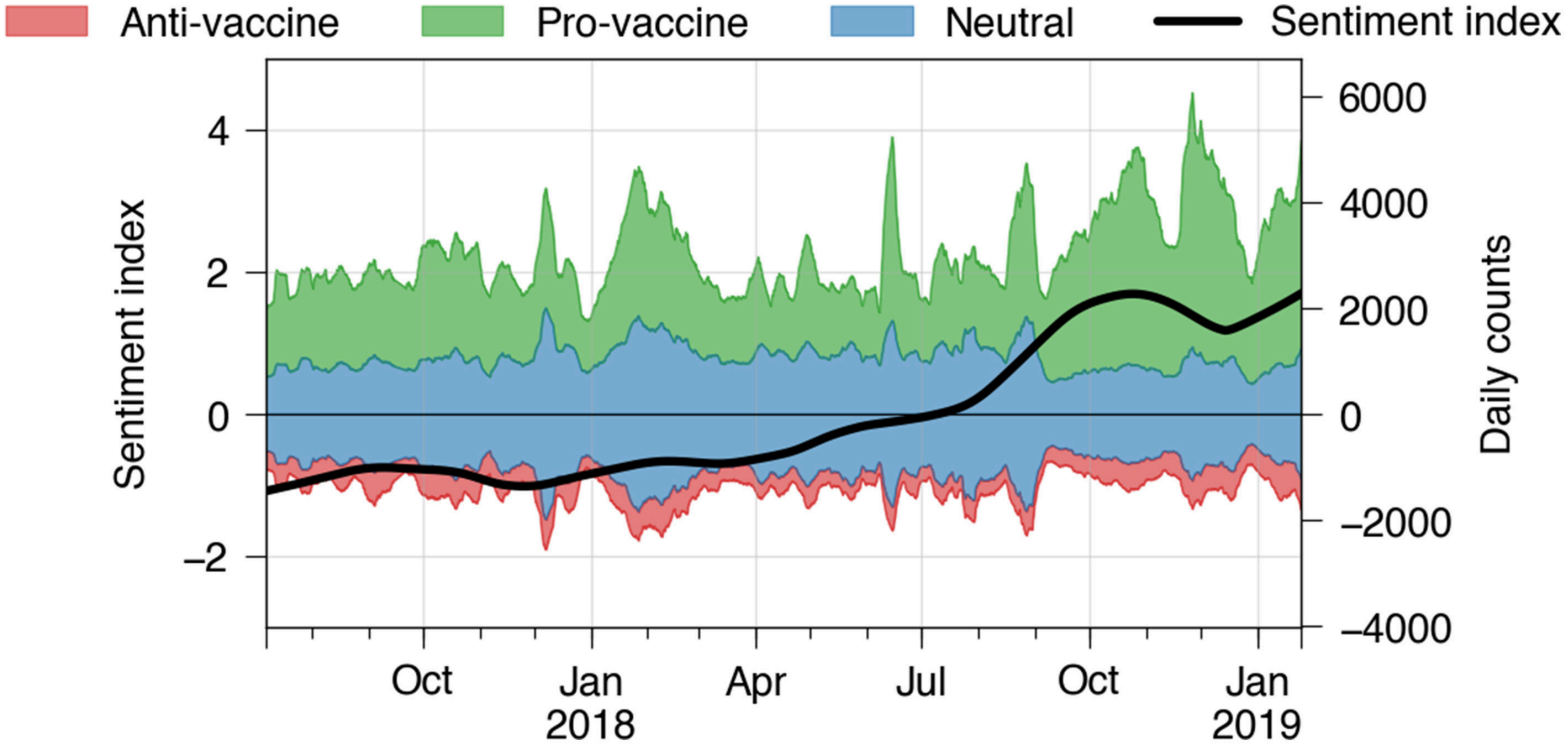
Real-time predictions of vaccine sentiments using Crowdbreaks. The data is based on a Twitter data stream filtered by vaccine-related keywords. Colored values indicate the stacked 1-week moving averages of tweet counts of the respective label class. The black curve denotes a sentiment index which reflects a lowess fit of the normalized ratio of counts of tweets predicted as positive and negative, aggregated in a 1 month window. The sentiment index reveals certain long-term trends irrespective of the high variance in volume over time.

Between July 2018 and January 2019 tweets were collected through the Twitter Streaming API using a list of vaccine-related keywords^5^ and predicted using a supervised bag-of-words fastText classifier^6^. The classifier was trained on annotated data (collected through MTurk) provided in recent work by Pananos et al. (29), resulting in micro-averaged precision and recall scores of 77.0%. The collected annotations include the label classes “positive,” “negative,” and “other” (in this work denoted as “neutral”) with regard to the attitude toward vaccinations the tweets express. For a detailed reasoning of how and why these specific labels and keywords were selected, please refer to the work by Pananos et al. As shown in Figure 4, we observe most of the discussion surrounding vaccination to be either neutral or positive. The fraction of data classified as “anti-vaccine” is below 10% and remains relatively constant at that level. Furthermore, we observe that the weekly tweet count exhibits a large variance in terms of volume over time. This effect can be mitigated by calculating a normalized ratio of positive and negative counts in a rolling window of 1 month, which we call “sentiment index” in Figure 4 (black curve). The sentiment index is calculated as (*r*−μ)/σ, in which *r* is the fraction of tweets predicted as positive among positive and negative tweets, and μ and σ are the mean and standard deviation of this ratio, respectively. This value remains largerly constant over time and then increases after August 2018, due to an increase in the number of tweets predicted as “pro-vaccine” and stays at that level. Further investigation will be needed in order to understand the nature of this change. Although these results are only of preliminary nature they illustrate the potential of the platform to track health trends over time.

## 5. Discussion

Here we introduced Crowdbreaks, an open tool allowing any researcher to start measurements of health trends in real-time from public social media content. As illustrated in the use case on vaccine sentiments, the platform can be used to monitor such sentiments and detect long-term shifts in health trends. Further analysis will be needed in order to reveal spatial sentiment distributions of the predicted vaccine sentiment as well as the correlation with vaccination coverage or disease outbreak data. Such analysis would however go beyond the scope of this work. Unlike in traditional settings of measuring vaccine sentiment, the platform involves crowdworkers as well as the general public to collect new annotations continuously over time. This allows to re-train models and counteract the problem of concept drift. In the future, we may use the platform to measure more fine-grained categorizations of this data, hence improving our understanding of attitudes toward vaccination.

A major goal of the platform is the eventual incorporation of similar models into the public health decision-making process. In order to achieve this, there is a need for proper validation and benchmarking of machine learning models, which in turn increases both trust and transparency of algorithms used for such purpose (31). In the future, annotation data generated on Crowdbreaks may be released in public challenges, thereby creating an open benchmark for a specific problem.

Although the platform focuses on the measurement of health trends, Crowdbreaks may also be used with regard to tracking flu or other infectious diseases in the future. However, disease prediction solely from Twitter data remains to be a hard problem. This is due to the fact that a precise understanding of the content (e.g., whether a tweet just raises awareness vs. actually reporting an infection) is crucial for the robustness of the model. Previous work has suggested hybrid models between Twitter and less volatile data sources (such a Wikipedia page rate clicks) to be superior for the purpose of outbreak tracking (32, 33). Such hybrid models may serve as a future direction for disease prediction projects on Crowdbreaks.

## Author Contributions

MM built the platform, did the analysis and wrote large parts of the papers and made the figures. MS had the initial idea for the project, drafted the initial design of the platform and wrote the abstract of the paper. All authors revised the manuscript and made corrections.

### Conflict of Interest Statement

The authors declare that the research was conducted in the absence of any commercial or financial relationships that could be construed as a potential conflict of interest.

## Footnotes

^1^https://www.mturk.com

^2^https://www.crowdbreaks.org

^3^https://github.com/crowdbreaks/crowdbreaks-streamer

^4^https://github.com/crowdbreaks/crowdbreaks

^5^The keywords include “vaccine”, “vaccination”, “vaxxer,” “vaxxed,” “vaccinated,” “vaccinating,” “vacine”.

^6^Data and code of the analysis are provided under https://github.com/salathegroup/crowdbreaks-paper.

## References

[1] Culotta A Towards detecting influenza epidemics by analyzing Twitter messages. In: Proceedings of the First Workshop on Social Media Analytics. Washington, DC: ACM (2010). p. 115–22.

[2] Paul MJ Dredze M You are what you Tweet: analyzing Twitter for public health. Icwsm. (2011) 20:265–72. Retrieved from: https://www.aaai.org

[3] Salathé M Khandelwal S. Assessing vaccination sentiments with online social media: implications for infectious disease dynamics and control. PLoS Comput Biol. (2011) 7:e1002199. 10.1371/journal.pcbi.1002199 22022249 PMC3192813

[4] Paul MJ Dredze M A model for mining public health topics from Twitter. Health. (2012) 11:16–6. Retrieved from: https://www.semanticscholar.org

[5] Parker J Wei Y Yates A Frieder O Goharian N A framework for detecting public health trends with twitter. In: Proceedings of the 2013 IEEE/ACM International Conference on Advances in Social Networks Analysis and Mining. Niagara Falls, ACM (2013). p. 556–63.

[6] Widmer G Kubat M Learning in the presence of concept drift and hidden contexts. Mach Learn. (1996) 23:69–101. 10.1007/BF00116900

[7] Ginsberg J Mohebbi MH Patel RS Brammer L Smolinski MS Brilliant L. Detecting influenza epidemics using search engine query data. Nature. (2009) 457:1012. 10.1038/nature07634 19020500

[8] Lazer D Kennedy R King G Vespignani A. The parable of google flu: Traps in big data analysis. Science. (2014) 343:1203–5. 10.1126/science.12485062014 24626916

[9] Butler D. When Google got flu wrong. Nature. (2013) 494:155–6. 23407515 10.1038/494155a

[10] Simpson R Page KR De Roure D Zooniverse: observing the world's largest citizen science platform. In: Proceedings of the 23rd International Conference on World Wide Web. (2014). p. 1049–54. 10.1145/2567948.2579215

[11] Crowdcrafting Available online at: https://crowdcrafting.org

[12] Wood C Sullivan B Iliff M Fink D Kelling S. eBird: engaging birders in science and conservation. PLoS Biol. (2011) 9:e1001220. 10.1371/journal.pbio.1001220 22205876 PMC3243722

[13] Khatib F Dimaio F Cooper S Kazmierczyk M Gilski M Krzywda S. Crystal structure of a monomeric retroviral protease solved by protein folding game players. Nat Struct Mol Biol. (2010) 18:1175–7. 10.1038/nsmb.2119 21926992 PMC3705907

[14] Russakovsky O Deng J Su H Krause J Satheesh S Ma S ImageNet large scale visual recognition challenge. Int J Comput Vis. (2015) 115:211–52. 10.1007/s11263-015-0816-y

[15] Kovashka A Russakovsky O Fei-Fei L Grauman K Crowdsourcing in computer vision. Foundations and Trends® in computer graphics and Vision. (2016) 10:177–243. 10.1561/0600000073

[16] Welinder P Perona P Online crowdsourcing: rating annotators and obtaining cost-effective labels. In: 2010 IEEE Computer Society Conference on Computer Vision and Pattern Recognition-Workshops, San Francisco, CA: CVPRW (2010). p. 25–32.

[17] Ho CJ Vaughan JW Online task assignment in crowdsourcing markets. In: Proceedings of the Twenty-Sixth AAAI Conference on Artificial Intelligence. (2012) (Kuhn 1955). p. 45–51. Available online at: http://arxiv.org/abs/1508.03593

[18] Hughes AJ Mornin JD Biswas SK Bauer DP Bianco S Gartner ZJ Quantius: generic, high-fidelity human annotation of scientific images at 10^5-clicks-per-hour. Nat Methods. (2017). 10.1038/s41592-018-0069-0

[19] Bengio Y Ducharme R Vincent P Janvin C A neural probabilistic language model. J Mach Learn Res. (2003) 3:1137–55. 10.1162/153244303322533223

[20] Mikolov T Chen K Corrado G Dean J Efficient estimation of word representations in vector space. arXiv [preprint]. arXiv:13013781 (2013). Retrieved from: https://arxiv.org

[21] Joulin A Grave E Bojanowski P Mikolov T Bag of tricks for efficient text classification. arXiv [preprint]. arXiv:160701759 (2016). Retrieved from: https://arxiv.org

[22] Deriu J Lucchi A De Luca V Severyn A Müller S Cieliebak M Leveraging large amounts of weakly supervised data for multi-language sentiment classification. In: Proceedings of the 26th International Conference on World Wide Web. International World Wide Web Conferences Steering Committee (2017). p. 1045–52.

[23] Kholghi M De Vine L Sitbon L Zuccon G Nguyen A Clinical information extraction using small data: an active learning approach based on sequence representations and word embeddings. J Assoc Inf Sci Technol. (2017) 68:2543–56. 10.1002/asi.23936

[24] Zhang Y Wallace B Active Discriminative Word Embedding Learning. NAACL (2016). Available online at: http://arxiv.org/abs/1606.04212

[25] Seeman N Ing A Rizo C. Assessing and responding in real time to online anti-vaccine sentiment during a flu pandemic. Healthc Q. (2010) 13:8–15. 10.12927/hcq.2010.21923 20959725

[26] Larson HJ Ghinai I. Lessons from polio eradication. Nature. (2011) 473:446. 10.1038/473446a 21614056

[27] Yahya M Polio vaccines— “no thank you!” barriers to polio eradication in Northern Nigeria. Af Aff. (2007) 106:185–204. 10.1093/afraf/adm016

[28] Larson HJ Smith DMD Paterson P Cumming M Eckersberger E Freifeld CC. Measuring vaccine confidence: analysis of data obtained by a media surveillance system used to analyse public concerns about vaccines. Lancet Infect Dis. (2013) 13:606–13. 10.1016/S1473-3099(13)70108-7 23676442

[29] Pananos AD Bury TM Wang C Schonfeld J Mohanty SP Nyhan B. Critical dynamics in population vaccinating behavior. Proc Natl Acad Sci USA. (2017) 114:13762–7. 10.1073/pnas.1704093114 29229821 PMC5748162

[30] Bahk CY Cumming M Paushter L Madoff LC Thomson A Brownstein JS. Publicly available online tool facilitates real-time monitoring of vaccine conversations and sentiments. Health Aff. (2016) 35:341–7. 10.1377/hlthaff.2015.1092 26858390

[31] Salathé M Wiegand T Wenzel M Focus group on artificial intelligence for health. arXiv [preprint]. arXiv:180904797 (2018). Retrieved from: https://arxiv.org

[32] McIver DJ Brownstein JS. Wikipedia usage estimates prevalence of influenza-like illness in the united states in near real-time. PLoS Comput Biol. (2014) 10:e1003581. 10.1371/journal.pcbi.1003581 24743682 PMC3990502

[33] Santillana M Nguyen AT Dredze M Paul MJ Nsoesie O Brownstein JS. Combining search, social media, and traditional data sources to improve influenza surveillance. PLoS Comput Biol. (2015) 11:e1004513. 10.1371/journal.pcbi.1004513 26513245 PMC4626021

